# An Unusual Presentation of a Patient With Low-Dose Methotrexate Causing Colitis and Pancytopenia

**DOI:** 10.7759/cureus.33062

**Published:** 2022-12-28

**Authors:** Ramya R, Nikola Malic, Trishala Menon, Edward Marks, Sathyanarayana Machani

**Affiliations:** 1 Family Medicine, WVU Medicine Wheeling Hospital, Wheeling, USA; 2 Family Medicine, WVU Medicine Uniontown Hospital, Uniontown, USA

**Keywords:** symptomatology, rheumatoid arthritis, pancytopenia, colitis, methotrexate

## Abstract

This paper summarizes the case of a patient admitted to the hospital due to methotrexate-induced colitis in the setting of pancytopenia. Although methotrexate is commonly used for a variety of diseases, this medication can precipitate side effects in a subset of patients. This is an atypical case of chronic low-dose methotrexate use leading to colitis and pancytopenia. It is of utmost significance to monitor patients on methotrexate through routine laboratory checks such as complete blood counts and liver function tests to ensure safety and reduce mortality.

## Introduction

Methotrexate is a widely used drug to treat a variety of diseases including but not limited to malignancies, skin conditions such as psoriasis, ectopic pregnancies, inflammatory bowel diseases such as Crohn’s disease, and a wide group of autoimmune diseases such as rheumatoid arthritis [1]. Even when used in low doses, chronic use of methotrexate can lead to several side effects that are often not noticed until a patient is hospitalized or critically ill. This case report is about a unique presentation of a patient on low-dose methotrexate for rheumatoid arthritis which led to pancytopenia and colitis.

## Case presentation

The patient is a 75-year-old Caucasian female with a past medical history significant for rheumatoid arthritis, hyperlipidemia, and controlled diabetes mellitus type 2 and presented to the emergency department with a four-day history of generalized weakness, poor oral intake, dyspnea on exertion, multiple episodes of non-bloody diarrhea, right lower quadrant abdominal pain, and epistaxis. Medication history includes metformin 1000 mg twice daily, ferrous sulfate 325 mg once daily, methotrexate 2.5 mg once daily, aspirin 81 mg once daily, and albuterol 90 mcg as needed. She denied having any fever, chills, night sweats, shortness of breath, or chest pain. Four months prior to her presentation, her methotrexate oral dosage was changed from 20 mg once weekly to 2.5 mg once daily due to worsening rheumatoid arthritis symptoms. On admission, the patient was afebrile, with a normal heart rate, resting comfortably on room air, However, she was hypotensive with BP 91/51 mmHg. Her physical examination was significant for diffuse abdominal tenderness to palpation. She had no petechiae, conjunctival pallor, or hepatosplenomegaly. No signs of peritoneal irritation were noted.

**Figure 1:**
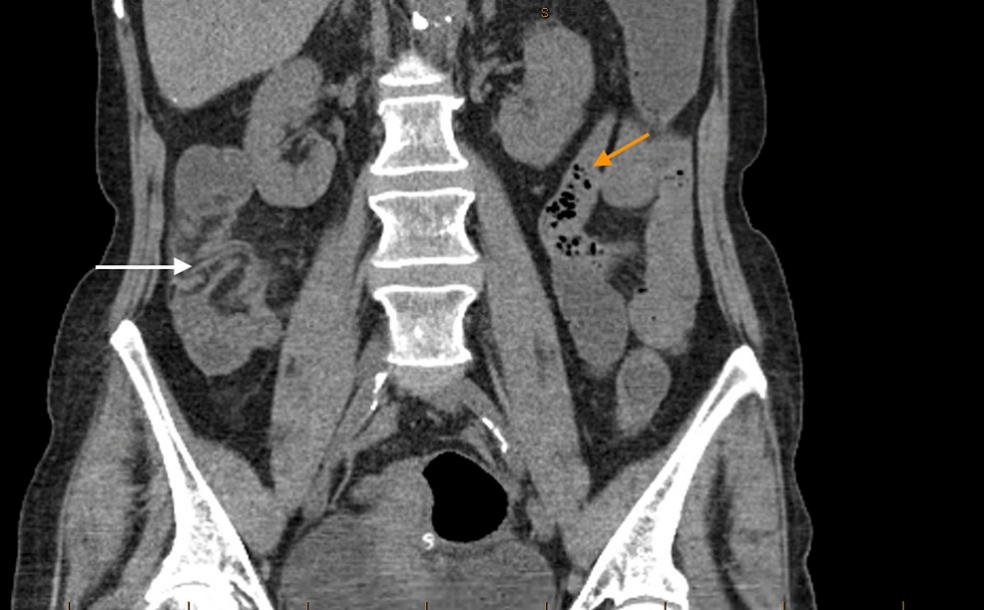
CT scan of abdomen and pelvis without oral contrast: colonic ileus with air fluid levels (shown with an orange arrow) along with colitis within the cecum and ascending colon (shown with a white arrow). CT, computed tomography

Her initial laboratory data revealed pancytopenia. She had a white blood cell count of 3.2 x 109 per liter, (neutrophils = 77.8%, lymphocytes = 20.4%, basophils = 0.6%, and eosinophils = 1.1%), a red blood cell count of 2.23 x 109 per liter, a hemoglobin of 7.3 gram/deciliter, reticulocytes of 19.7 k/ul, and a platelet count of 13 x 109 per liter (Table 1). She also had acute hyponatremia (sodium of 128 milliequivalents/liter) and acute kidney injury (creatinine of 3.32 milligram/deciliter with a baseline creatinine of 1.1 milligram/deciliter. Her serum liver enzymes (AST 25, ALT 19, alkaline phosphatase 100) and vitamin B12, folate, albumin, and lactate levels were all within normal limits. She also had elevated inflammatory markers with a c-reactive protein of 5.9 milligram/deciliter, an erythrocyte sedimentation rate > 140 millimeter/hour, and a lactate dehydrogenase of 259 microliter. Blood cultures were negative. Her enteric pathogen panel and Clostridium difficile were negative. She also had negative cytomegalovirus and Epstein Barr virus panels. Her computed tomography scan of abdomen and pelvis without oral contrast showed colonic ileus with air fluid levels along with colitis within the cecum and ascending colon (Figure 1). Her abdominal ultrasound and urinalysis were negative.

**Table 1:** Trajectory of pancytopenia before and after filgrastim administration Filgrastim therapy initiated on Day 3 Completed three days of filgrastim on Day 6 Discharged on Day 13 with resolution of symptoms WBC, white blood cell; RBC, red blood cell

Parameter	Baseline	Day 1	Day 3	Day 6	Day 11	Follow-up clinic 10 days post discharge
WBC	11	3.2	1.2	4.4	9.5	12.9
RBC	3.5	2.23	2.9	2.9	2.35	2.66
Platelets	330000	13000	55000	86000	362000	765000
Hemoglobin	10	7.3	9.4	9.3	7.2	8.5
Creatinine	1.12	3.32	2.41	1.04	1.05	1.1

 

She received a three-day course of filgrastim 300mg subcutaneously, two units of packed red blood cells, and one unit of platelets. She initially received broad-spectrum antibiotics: cefepime and metronidazole. They were given prophylactically for a suspicion of infectious colitis with severe sepsis criteria being met. However, her broad-spectrum antibiotics were later discontinued with no clear infectious etiology being identified. She was symptomatically improved with steady increases in all cell lines seen with methotrexate being held on days 6 to 11 of admission. Her acute kidney injury resolved with intravenous hydration. She was discharged on day 13 of admission when her cell counts returned to normal and diarrhea had resolved. Naranjo criteria is a method for estimating the probability of adverse drug reactions. This method was used to determine the strength of this supposed drug reaction and patient's score was 8 which indicated a probable drug reaction. It is evident from this patient’s hospital course that methotrexate led to her colitis and pancytopenia. The patient had significant improvement in her clinical status when methotrexate was held. The patient refused colonoscopy; therefore, no histopathologic or colonoscopies findings were available. 

It was not noted whether the patient was made aware that methotrexate could have led to such adverse reactions. The patient was not educated about the monitoring required while being on methotrexate. Tests such as complete blood counts and liver enzymes that are typically needed to monitor while a patient is on methotrexate are essential presenting practice. 

## Discussion

As presented above, this is a case of severe colitis and pancytopenia secondary to low-dose methotrexate. The evidence of colitis on imaging in the setting of pancytopenia is uncommon and yet a deadly complication. This complication also raises the concern for possible serious opportunistic infections such as bacterial and viral colitis [1,2]. Diarrhea in this particular case cannot be attributed to medications such as semaglutide and/or atorvastatin as the patient was not on these medications. Her diabetes mellitus was controlled since her recent hemoglobin A1C was 6.9 prior to the admission. It is a known fact that metformin can cause diarrhea in a certain subset of patients. However, she has been on metformin for about 20 years and has never complained of being intolerant of gastrointestinal side effects. 

It is a known fact that methotrexate has side effects like other medications. However, it is a lesser-known fact that methotrexate toxicity can occur in low doses. Therefore, physicians need to be prompt in recognition of adverse events. 

Methotrexate-induced adverse events are not very common and are not well studied or documented. These adverse events can occur in a small subset of methotrexate-prescribed patients. This group needs to be closely monitored. Methotrexate is a widely used medication however its adverse reactions are not all commonly noticed. Most patients on methotrexate are made aware that they need regular lab tests to detect adverse outcomes such as pancytopenia. The rate of occurrence of colitis with methotrexate use has not been documented because of its rarity. More cases of methotrexate-induced non-infectious colitis need to be identified. It is worth noting that colonoscopies would be essential in patients with methotrexate-induced colitis to identify any associated histopathologic findings. 

Primary care physicians must spend some time in educating patients regarding the necessary monitoring and the adverse reactions associated with methotrexate therapy. They also must evaluate the patients regularly for signs and symptoms of potential toxicity [3]. Regular lab measurements of methotrexate-treated patients including complete blood counts and liver function tests can avoid adverse outcomes such as pancytopenia and liver dysfunction [2].

In addition to pancytopenia, another less known side effect of methotrexate is colitis. Even patients currently receiving low doses of methotrexate such as 6 mg per week have developed colitis [4].

In rare instances of methotrexate-induced colitis, folic acid has been shown to lead to resolution of symptoms. Co-administration of folinic acid and granulocyte colony-stimulating factors has been the best response to methotrexate-induced colitis [5].

It is essential prior to initiation of methotrexate therapy to investigate for any underlying liver or kidney dysfunction as this could have fatal outcomes. The most important from a primary care physician perspective would be to ensure that patients understand any adverse outcomes, recognition of symptoms that warrant seeking emergent medical assistance, and emphasizing the significance of regular laboratory monitoring and follow-up appointments. 

MTX is a very commonly used drug in rheumatology, knowing all the side effects including the rare ones is important. Due to scarcity of resources, there are several unanswered questions as to when methotrexate can be re-introduced in these subsets of patients with colitis. Therefore, screening for methotrexate toxicity including rare adverse events is an important tool in hospital medicine. 

## Conclusions

Although an effective treatment strategy for rheumatoid arthritis, low-dose methotrexate therapy can be implicated in many adverse effects as seen in our case. Prompt recognition of methotrexate toxicity in low doses is essential in preventing further and more deadly complications associated with pancytopenia and colitis. The patient gave informed consent for the study which was signed and documented. 
